# The *Regeneración Urbana, Calidad de Vida y Salud* - RUCAS project: a Chilean multi-methods study to evaluate the impact of urban regeneration on resident health and wellbeing

**DOI:** 10.1186/s12889-021-10739-3

**Published:** 2021-04-15

**Authors:** Fernando Baeza, Alejandra Vives Vergara, Francisca González, Laura Orlando, Roxana Valdebenito, Andrea Cortinez-O’Ryan, Claire Slesinski, Ana V. Diez Roux

**Affiliations:** 1 Department of Public Health, Pontificia Universidad Católica de Chile, Diagonal Paraguay 362, 8330077 Santiago, Chile; 2 Institute of Geography, Pontificia Universidad Católica de Chile, Vicuña Mackenna 4860, 7820436 Santiago, Chile; 3 Centre for Sustainable Urban Development (CEDEUS), Los Navegantes 1963, 7520246 Santiago, Chile; 4 Department of Physical Education, Sports and Recreation, Universidad de La Frontera, Moneda 673, 8320216 Santiago, Chile; 5 Urban Health Collaborative, Dornsife School of Public Health, Drexel University, 3215 Market St, Philadelphia, PA 19104 USA

**Keywords:** Urban regeneration, Prospective longitudinal study, Urban health, Latin America, Housing policy, Natural experiment, Neighborhood renewal, Housing improvement

## Abstract

**Background:**

The available evidence of the health effects of urban regeneration is scarce In Latin America, and there are no studies focused on formal housing that longitudinally evaluate the impact of housing and neighborhood interventions on health. The “*Regeneración Urbana, Calidad de Vida y Salud*” (Urban Regeneration, Quality of Life, and Health) or RUCAS project is a longitudinal, multi-method study that will evaluate the impact of an intervention focused on dwellings, built environment and community on the health and wellbeing of the population in two social housing neighborhoods in Chile.

**Methods:**

RUCAS consists of a longitudinal study where inhabitants exposed and unexposed to the intervention will be compared over time within the study neighborhoods (cohorts), capitalizing on interventions as a natural experiment. Researchers have developed a specific conceptual framework and identified potential causal mechanisms. Proximal and more distal intervention effects will be measured with five instruments, implemented pre- and post-interventions between 2018 and 2021: a household survey, an observation tool to evaluate dwelling conditions, hygrochrons for measuring temperature and humidity inside dwellings, systematic observation of recreational areas, and qualitative interviews. Survey baseline data (956 households, 3130 individuals) is presented to describe sociodemographics, housing and health characteristics of both cohorts, noting that neighborhoods studied show worse conditions than the Chilean population.

**Discussion:**

RUCAS’ design allows for a comprehensive evaluation of the effects that the intervention could have on various dimensions of health and health determinants. RUCAS will face some challenges, like changes in the intervention process due to adjustments of the master plan, exogenous factors –including COVID-19 pandemic and associated lockdowns– and lost to follow-up. Given the stepped wedge design, that the study capitalizes on within household changes over time, the possibility of adjusting data collection process and complementarity of methods, RUCAS has the flexibility to adapt to these circumstances. Also, RUCAS’ outreach and retention strategy has led to high retention rates. RUCAS will provide evidence to inform regeneration processes, highlighting the need to consider potential health effects of regeneration in designing such interventions and, more broadly, health as a key priority in urban and housing policies.

**Supplementary Information:**

The online version contains supplementary material available at 10.1186/s12889-021-10739-3.

## Background

There is strong evidence that housing conditions and the built and social environments of neighborhoods affect health [1–8]. These effects involve a complex set of interrelated causal processes in which specific housing and environment features may be related to multiple outcomes [8].

Health relevant features of built and social environments include the availability of green space and healthy foods [9, 10], social capital and violence [11–13]; and housing and household-related factors such as overcrowding [14], thermal comfort [15, 16] or dampness and mold [17]. Strong evidence has shown the impact of these environments on specific health behaviors and outcomes such as physical activity, diet and obesity [18–20], respiratory health [16, 21], and general physical and mental health [6, 16, 21], among others.

Within the framework of urban health, researchers posit that unequal access and exposure to the benefits and risks of urban life are fundamental social determinants of health inequalities. In large Latin American cities, the spatial distribution of populations follows a clear pattern of inequality as a product of exclusion and gentrification at both the level of cities and neighborhoods [22, 23], causing populations to cluster geographically, based on socioeconomic characteristics that are also linked to health inequities [24, 25]. One of the drivers of such inequalities in Chile has been the social housing policy in place since the 1980s, which has created a segregated periphery, especially, in large metropolitan cities. As a result of segregation, both individual-level socioeconomic characteristics and health are spatially clustered, and substantial differences across neighborhoods emerge, such as major inequalities in life expectancy [26, 27]. Although place and individual characteristics can be conceptualized as distinct, they are closely interrelated, because “there is a mutually reinforcing and reciprocal relationship between people and place” [28]. This relational perspective of place effects on health is particularly relevant in the study of marginalized or excluded neighborhoods [23].

While one solution to this problem is the mobility of low-income neighborhood dwellers, another, arguably more sustainable solution is the renovation and improvement of these neighborhoods. Several studies have evaluated whether improvements in housing and neighborhood conditions yield health benefits. Capitalizing on urban regeneration initiatives as natural experiments, these studies have shown increases in satisfaction with housing, improvements in perceived quality of life, reduction of violence, and mental health benefits, among others [29–33]. However, most of these studies have failed to support definitive conclusions regarding associations between urban regeneration and health [4, 34, 35]. Furthermore, most studies evaluating the health impacts of these interventions have been conducted in high-income countries. Studies implemented in low or middle-income countries such as those in Latin America have focused on interventions moving residents from slums to formal settlements with basic infrastructure (water, sanitation, solid waste collection, electricity) and have focused on communicable disease outcomes [30, 35–38]. As far as we know, there are no documented longitudinal studies that assess the impact of urban regeneration of formal social housing neighborhoods on health and wellbeing in Latin America.

By means of the evaluation of an urban regeneration program in Chile, the RUCAS Project (*Regeneración Urbana, Calidad de Vida y Salud,* or *Urban Regeneration, Quality of Life and Health* in English) aims to contribute to the evidence of how housing and built environment interventions may impact health and wellbeing.

This paper describes the RUCAS project, the intervention and neighborhoods studied, the data collection instruments and the RUCAS sample. In the rest of the Introduction, the intervention is described, and the conceptual framework is presented. The methods section details the various strategies for data collection, and the RUCAS sample is described based on the survey baseline data collection. The discussion section outlines the most significant challenges identified for the evaluation’s success and the approaches implemented to deal with them.

### Setting and interventions

As in most countries in Latin America, after an intensive urbanization process in the late twentieth century, Chile (according to Gini index, the second most unequal of the high-income countries globally [39]) faced a severe housing deficit with thousands of poor families living in informal settlements and slums known as “*tomas*” and “*campamentos*” [40]. In the late 1970s, the military dictatorship neoliberal government implemented social housing policies to address this formal housing deficit via the private sector. Between 1980 and 2000, the massive construction of 202,000 households [41] in “*villas*” of mid-rise three and four-story housing blocks within most medium-sized and large Chilean cities, proved to be a significant achievement in terms of housing coverage as a solution to a “quantitative” housing deficit [36]. The constructed apartments were then provided at very low or no cost to people living in poverty or extreme poverty. Currently, over one million people live in these neighborhoods [41].

These housing blocks are built in brick masonry and equipped with basic utilities (i.e., electricity, piped water, and connection to a sewage network). Eventually, this public housing solution proved incapable of meeting minimum living standards due to poor construction and rapid deterioration [40]. According to the Ministry of Housing (MINVU), more than half of these neighborhoods are currently in a situation of “high vulnerability” to deterioration [41] and present serious issues regarding lack of or poor quality public spaces, poor accessibility, high residential density, inferior building materials, insufficient acoustic and thermal insulation, insufficient living space (resulting in illegal self-built expansions), and considerable damage to utilities [36, 40].

Following the acknowledgement of the poor quality of these housing blocks, housing policies shifted their attention towards the “qualitative” deficit of buildings and their surrounding neighborhoods, initially through massive demolition of buildings and relocation of neighbors (*Programa Segunda Oportunidad*), without consideration of best practices for urban planning and design. Today, however, following guidelines developed by United Nations as described in their New Urban Agenda [42], MINVU’s focus is on urban regeneration of these housing blocks and neighborhoods, aiming to implement the restoration and redevelopment of physical and social environments in urban areas that have experienced economic and environmental decline [43]. This alternative has proved to be more environmentally sustainable while also comprehensively addressing the needs of families living in these homes [44] while encouraging cooperation with local communities.

MINVU’s *Programa de Regeneración de Conjuntos Habitacionales* (“the intervention”), initiated in 2013, aims to “regenerate social housing complexes whose urban and residential configuration has insufficient or deteriorated access, roads, green space, and facilities, weakened community organizations and deficiencies in the size and habitability of housing” [45]. Overall, 172 neighborhoods (306,000 inhabitants) meet the conditions to be beneficiaries of this program, although it is currently being implemented in only 11 [45]. The study described here is being implemented in two of these neighborhoods where the period of planned intervention coincided with the time frame of the study: “BDM”, which is located in the northern periphery of the city of Viña del Mar, where 384 homes built in 1992 will be intervened on; and “MB”, in the southern periphery of the city of Santiago, where 1256 homes built in 1996 will be intervened on (see Table 1).

**Table 1:** RUCAS neighborhoods and primary interventions by component

		BDM	MB
Overall neighborhood description	Year of construction	1992	1996
	Travel time to the city’s downtown by public transport	25 min	70 min
	Inhabitants (census 2017)	831	3834
	Number of dwellings pre-intervention	384	1256
Housing interventions	Expansion: Dwelling area (m^2^) before the intervention	42	42
	Expansion: Dwelling area (m^2^) after the intervention	57	84
	Thermal and acoustic insulation	yes	yes
	Improvement of roofs	yes	yes
	Upgrading or installation of utilities (sanitation, electrical)	yes	yes
Final housing location	Remodeled but not expanded dwelling within the villa	yes	yes
	Same remodeled dwelling	yes	yes
	Different rehabilitated dwelling within the villa	yes	yes
	New dwelling within the villa	yes	yes
	New dwelling in an adjacent new villa	no	yes
	Undetermined dwelling outside the villa (expropriation)	yes	yes
Built environment interventions	Dwellings demolished (as % of existing dwellings before intervention)	18%	34%
	New recreational public spaces (green areas, parks)	yes	yes
	Sports facilities (sport courts, playgrounds)	no	yes
	Tree planting on streets and in parks	yes	yes
	Improvement of roads (streets, sidewalks)	yes	yes
	Improvement of existing public lighting	yes	yes
	New bus stop	no	yes
Community interventions	New community centers	yes	no
	Participatory social diagnoses	yes	yes
	Revitalization of community organizations	yes	yes

The intervention is comprehensive, with a master plan developed for each villa detailing the content of the planned works which may vary according to the needs of each neighborhood. The intervention can be disaggregated into three components:

1. Housing: The expansion and remodeling of dwellings

The intervention aims to meet current standards for the construction of social housing, which are now improved as compared to those of the 1990s [45]. As can be seen in Table 1, different solutions will be provided depending on preference (stay or leave the villa) and feasibility. Most dwellings will be expanded (in BDM) or merged (in MB), growing from 42 to 57 m^2^ in BDM, and from 42 to 84 m^2^ in MB, on average. In all dwellings, equipment will be replaced, and new insulation throughout the building walls will be provided. While in MB the extension works are carried out with the families out of the apartments (they must leave for approximately 1 year and are temporarily placed in other apartments that may be inside or outside the villa), in BDM the works are carried out along the perimeter of the buildings, with the families living inside while construction is done, which may last from 6-8 months.

2. Built environment: Construction and improvement of parks, urban equipment, and roads, among others. Dwelling’s demolition.

This component includes the renovation and construction of new public spaces, green areas with sports facilities and playgrounds, replacement of street surfaces and sidewalks, and public lighting improvement. Also, to reduce residential density and provide space for new green areas, a small proportion of housing blocks were demolished previous housing intervention and baseline measurements. In BDM two new community centers and one new park were already in use when the study was initiated. In MB, park renovation will take place after data collection for this study has been initiated.

3. Community: Revitalization and strengthening of community organizations and neighborhood participation.

This component includes the implementation of participatory social diagnoses and reactivating neighborhood councils. The activities of this component, while fulfilling their own specific objectives, make the work of the other two components viable, thus accomplishing the mandate of the program to have a participatory design. These activities are implemented directly by MINVU professionals in each villa: *Secretaría Regional Ministerial V Región* (SEREMI) in BDM and *Equipo de Regeneración Urbana* (ERU) in MB.

In BDM, works associated with the intervention began in 2017 (before baseline), while in MB, the first works were carried out in 2019, after baseline data collection was completed. The differences in the number of households involved and the intervention strategies used in each villa mean that the intervention times are different, with work possibly extending until 2023 in BDM and 2028 in MB. In both cases, the interventions will be implemented within sections of the neighborhood in succession, adding new households to the group of those that have been “intervened” at each measurement wave (see Fig. 2 for a timeline of both planned interventions and data collection).

### The RUCAS project

The RUCAS Project aims to evaluate the effects of the intervention on both villas’ residents’ health and wellbeing, capitalizing on the intervention as a natural experiment. To this end, a prospective study has been designed to follow two cohorts (BDM and MB) over 4 years as the intervention unfolds, collecting primary data with repeated measures using five different data collection instruments. RUCAS is an ancillary study to the Salud Urbana en America Latina (SALURBAL) project [46].

The main research objectives addressed by the project include assessing the effect of dwelling improvements on general, mental, and respiratory health, and health-related outcomes; to estimate the impact of the improvement in recreational public spaces in the utilization of these areas and potential impact on general and mental health and perceptions regarding the neighborhood; and assess the impact of the intervention on dwellers perceptions and experiences.

### Conceptual framework

Based on the literature, we developed a conceptual model identifying the direct or proximal effects of each component of the intervention and the health outcomes most likely to be affected by these, considering seasonal variability in these effects. We focused on health effects that could be observed within the time frame of the project (i.e. short term effects) [29, 47, 48]. These are general health, respiratory and gastrointestinal health, mental health, and sleep quality. In addition, we identified relevant health-related outcomes that might be affected by the intervention such as health-related behaviors, household and neighborhood satisfaction, and family relations within the dwelling.

**Fig. 1:**
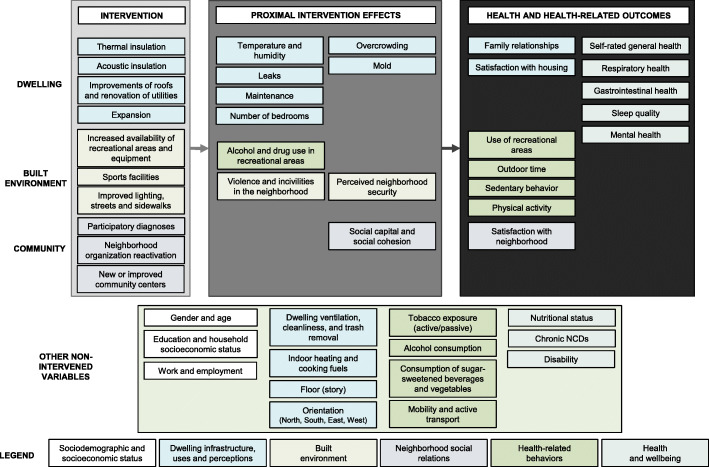
RUCAS analytic framework

Figure 1 describes this conceptual framework. On the left side, the main features of each component of the intervention are listed. The central section of the figure depicts the main direct or proximal effects of these components of the intervention. These, in turn, will be the determining factors of potential changes in health or health-related outcomes, listed on the box at the right. Key baseline characteristics and other intervening factors at the level of dwellings, households and individuals that may be relevant to better understand how the effects of the intervention are modified or vary across groups, are presented in the rectangle below. All elements of the conceptual framework are grouped into six domains, each represented by a different color: sociodemographics and socioeconomic status; dwelling habitability, uses and perceptions; built environment; neighborhood social relations; health-related behaviors; and health.

While the analytic framework separates the variables related to characteristics of dwellings, the built environment, and the community, may be challenging to distinguish how changes in health outcomes could be attributed separately to these three components (e.g., mental health could be improved by all three). On the other hand, it should be noted that the framework is a general approximation to the design of the instruments and analyses. Nevertheless, for each analysis, it will be necessary to evaluate the specific causal mechanisms that may be producing any observed outcomes.

## Methods

### Study design

**Fig. 2:**
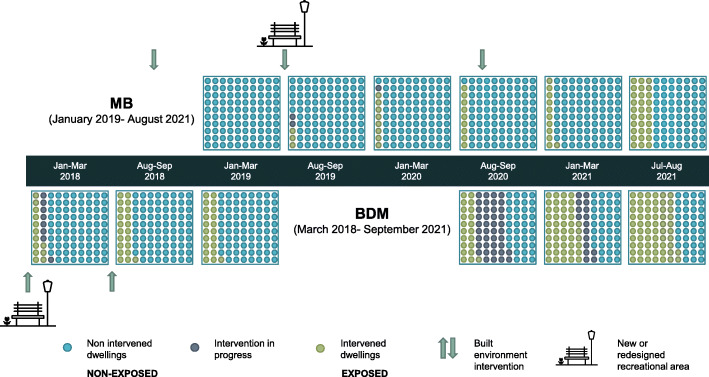
Programmed progress of the dwellings’ intervention status at each RUCAS survey wave, according to masterplan. Note: Each box represents a measurement wave, and each circle represents a 1 % of dwellings in the villa. The colors of the circles indicate the dwelling intervention status. Arrows represent the built environment interventions

Capitalizing on the urban regeneration program as a natural experiment, RUCAS is a longitudinal study where dwellers exposed and unexposed to the intervention will be compared over time within the study villas, following a stepped wedge design. At baseline, most dwellings had not yet been exposed, and by the final wave (winter 2021), nearly 30% in MB and 62% in BDM will have received the intervention. The progress of the intervention across dwellings and waves is shown in Fig. 2. In each villa, the housing intervention unfolds by groups of adjacent housing blocks, without expected systematic differences in the selection of which dwellings are intervened at each stage.

### Instruments and main variables

Five instrument sets were designed or adapted for this study. Each of these instruments will be applied at least twice (for intervened dwellings, pre- and post-intervention), according to the measurement timeline. A detailed representation of the proposed measurement timeline is presented in Additional file 1. Key domains, variables and measures are shown in Table 2.

1. Household survey (RUCAS survey): Face-to-face interviews administered by trained interviewers to one household key informant in their homes. Household key informants are over 18 years old and provide information concerning the household, dwelling and neighborhood, as well as health and health related data for themselves and each household member. The key informant is aged 18 years or older and, preferably, the female head of each household (*dueña de casa*), who is most likely to be at home at the time of the interview and to be better informed about the dwelling and the health and health-related conditions of family members. Three waves of the survey are programmed to be applied in summer (January–March) and three in winter (August–September), 6 waves in total in each villa (see Additional file 1). During the first and last summer wave, a “full-version” of the survey is planned, while in the rest of waves a “short-version” will be applied, measuring the main health and health-related outcomes and key time-varying characteristics, always to the same household key informant.
2. Intra-domiciliary observation tool (RUCAS IDO tool): Structured guideline applied by the interviewers, accordingly trained, to assess dwellings’ features such as the number of rooms, maintenance, and presence of mold, among others. It is administered jointly with the questionnaire in each dwelling in all waves.
3. Intra-domiciliary hygrothermal conditions (*hygrochrons*): In a sub-sample of dwellings, temperature and humidity data-loggers will be installed in winter and summer at the beginning and the end of the study for 2-4 weeks before the RUCAS survey. The first winter and summer measurements have been performed in each villa between the winter of 2018 and summer of 2020 in sub-samples of 34 and 30 dwellings in BDM and MB, respectively. The iButton Hygrochron [61] and Hobo Hygrometer [62] are standalone devices that measure and record temperature and humidity in a protected memory unit. They will provide objective, continuous measures of temperature and relative humidity in one bedroom and living room to evaluate hygrothermal conditions and thermal comfort.
4. Qualitative methods: Semi-structured individual interviews and focus groups will be conducted separately with SEREMI-ERU professionals, residents, and community leaders. In 2018, a first round of four pre-intervention focus groups were held in both villas, in addition to 6 in-depth interviews in BDM with residents of intervened and non-intervened dwellings and two interviews with SEREMI-ERU professionals. Interviews explore experiences and meanings associated with housing and the neighborhood, expectations regarding the intervention, and key aspects of the history of the villas and community organization.
5. Systematic observation of recreational areas: System for Observing Play and Recreation in Communities (SOPARC) [63] and Physical Activity Resource Assessment (PARA) [64] are planned to be applied in MB before and after interventions take place in recreational areas, and in an adjacent neighborhood as a control, selected considering similar size of the neighborhood, number of recreational areas, number of inhabitants and socioeconomic profile according to census data. Baseline measurement were carried out over 2 weeks in May of 2018. SOPARC uses systematic and periodic scans of individuals and contextual factors within pre-defined target areas, coding activity as sedentary, walking, or very active. These scans gather data separately for gender and various age groups. PARA is an assessment protocol to describe type, quantity and features of amenities, and quality of all publicly accessible physical activity resources in urban neighborhoods.

Most variables are collected periodically through one or more instruments. Key health outcomes are assessed with the RUCAS survey in various manners, some allowing for greater sensitivity, others greater specificity. For example, we measure respiratory health broadly, ranging from symptoms (more sensitive but less specific and prone to recall bias) to hospitalizations (granting higher specificity but less sensitivity and lower incidence). We also characterized dwelling-related exposures (e.g., thermal comfort) using both self-reports and objective measurements. In Table 2 we present the main study dimensions and the measurement instruments used to assess them.

**Table 2:** Study dimensions, main variables, and measurement instruments

Variable	RUCAS survey	RUCAS IDO tool	Hygro-chrons	Systematic observation	Interviews	SEREMI-ERU ^a^
**Dwelling habitability, uses and perceptions**						
Dwelling intervention status (insulation, improvement of roofs, renovation of utilities, and expansion)		X				X
Intra-domiciliary temperature and humidity (objective and perceived)	X		X			
Leaks and dwelling maintenance (state of conservation of walls, ceilings, and floor)	X	X				
Bedrooms per dwelling, overcrowding (residents per bedroom [49] and insufficiency of bedrooms [50]) and presence of illegal self-built expansions	X	X				
Mold (observed and smelled)	X	X				
Family relationships related to the use of dwelling	X					
Satisfaction with housing (amount of space, insulation of exterior noises, temperature, natural light availability). Experiences related to dwelling	X				X	
Dwelling ventilation, cleanliness, and trash removal. Indoor heating and cooking fuels	X	X				
Floor (story) and orientation of the main façade (North, South, East, West)						X
**Built environment**						
Neighborhood and community intervention status (recreational areas, equipment and sport facilities, improved lightning, streets, and sidewalks)				X		X
Violence and incivilities in the neighborhood [51]	X			X		
Perceived neighborhood security (informant perceptions in several situations at the neighborhood)	X					
**Neighborhood social relations**						
Community intervention status (participatory diagnoses, neighborhood organization and community centers). Perceptions about the intervention.					X	X
Social capital and social cohesion between neighbors (trust, reciprocity, and conflicts)	X					
Satisfaction with the neighborhood. Villas’ history and perceptions about changes	X				X	
**Health-related behaviors**						
Alcohol and drug use in recreational areas (number of visitors using drugs or consuming alcohol)				X		
Use of recreational areas: Number of courts and parks visitors by physical activity level (MB only), and courts and park visits (days a week)	X			X		
Outdoor time, physical activity (GPAQ [52]), sedentary behavior for adults (GPAQ) and screen-time for children [53]	X					
Active and passive smoking. Problematic alcohol consumption (AUDIT-dimension 1 [54]). Consumption of vegetables and sugar-sweetened beverages. Daily mobility and active transport	X					
**Health**						
Self-rated general health (7 points scale)	X					
Respiratory health (acute respiratory conditions, inhaler requirement, emergency department visits and hospitalizations)	X					
Gastrointestinal health (acute gastrointestinal symptoms (vomiting, diarrhea), emergency department visits and hospitalizations	X					
Sleep complaints (frequency of problems falling asleep, sleep disruption and daytime sleepiness)	X					
Mental health (GHQ-12 [55, 56] for the informant and PHQ-2 [57] for other householders over 15)	X					
Nutritional status. Medically diagnosed non-communicable diseases. Infant development (items from Parent’s Evaluation of Developmental Status, PEDS [58]) and school repetency. Disability and limitations to activities of daily living (items taken from the Barthel index) [59]	X					
**Sociodemographics and socioeconomic status**						
Gender and age	X					
Education and socioeconomic status (individual and household)	X					
Work and employment: labor force status, occupation, and employment precariousness (EPRES [60])	X					

^a^Information is provided by the professionals from the ministry of housing in Viña del Mar (SEREMI) and Santiago de Chile (ERU)

### Data collection, sampling strategy and follow up

In BDM, data gathering began after the dwelling intervention had begun and families not eligible for the intervention had already left, so we used a census strategy to recruit participants (i.e., key informants), visiting each dwelling in the villa. In MB we initiated data collection before the dwelling intervention began, so the sampling frame was provided by ERU in the form of a list of households eligible for intervention. In both villas, households representing security concerns, dwellings used for non-residential purposes, and dwellings without inhabitants were excluded (28 in BDM and 22 in MB). This resulted in a sampling frame of 260 dwellings in BDM and 807 in MB.

At each survey wave we contacted all households that we had contact (or attempted to contact) in the prior wave, except for refusals. Those who left the villa are contacted by telephone and surveyed one last time, and then dropped from the sample. Dwelling and neighborhood level data, as well as information on individual household members reported by the key informant yields a sample of individuals clustered within households. In successive waves, new members of households were included in the survey. When strictly necessary the original key informant has been replaced by another member of the household, due to physical or mental impairment that made him/her unable to answer the survey, death or prolonged absence.

### Outreach activities

Considering that lost to follow-up represents one of the greatest challenges in a longitudinal study, substantial efforts have been devoted to the follow-up of the participants of both cohorts. A team member who keeps regular contact with the villa’s community leaders, is fully dedicated to these activities. Based on recommendations identified in the literature [65], the team has maintained a permanent contact with the villas and participants, inviting them to answer the survey in each wave through hand-outs, posters and social media, including on them preliminary descriptive results. Participants not contacted in person during fieldwork have been contacted by telephone to find out if they live still in the dwelling or in the villa. For this reason, it has been fundamental to collect and update contact information regularly for each household, retrieving phone numbers for the respondent and at least one other contact person (relatives, friends, or neighbors) in each successive survey wave.

Both the study design as well as a selection of preliminary results have been systematically shared with community leaders and with SEREMI-ERU, not only for retention, but also for the validation of findings, and to provide valuable information for the interventions’ masterplans and recommendations for households. Regular meetings with SEREMI-ERU are held to maintain awareness among the research team of the evolution of the master plans for the two neighborhoods and to monitor the progress of the interventions in each villa. Preliminary results have also been presented to Ministry of Housing authorities on several occasions, emphasizing the need to evaluate housing policies and urban regeneration from a health perspective in the medium and long term.

### Planned analytical approach

The design of RUCAS allows for a broad range of cross-sectional and longitudinal hypotheses to be tested, drawing on repeated measures on exposed and unexposed groups. The impact of dwelling improvements on health will be assessed via multilevel models. Considering the stepped wedge design for this study [66], repeated measures over time are nested within households. This will allow us to estimate the effect of a time-varying intervention while adjusting for time varying and time invariant covariates (including temporal trends unrelated to the intervention and season -summer or winter wave).

The impact of the improvement of recreational public spaces on use of these spaces will be estimated using survey data for courts and park visits and by comparing changes in PARA and SOPARC results between the intervened (MB) and the control neighborhood, using a difference-in-difference analysis [67]. We will use qualitative content analysis [68], to describe the perceptions of inhabitants, the meanings of the intervention and changes in the narratives as well as to delve into the mechanisms that could lead to health improvements.

### RUCAS sample description

By July 2020, three waves of the RUCAS survey and IDO tool have been applied in each villa (see Additional file 1). In both villas combined, 956 households in 948 dwellings were interviewed at baseline, representing 88.8% of the dwelling target sample and including 3130 individuals. Key informants are aged between 18 and 90 years with a mean of 48.2 years, 74.1% reported 8 or more years of formal education, and, consistent with expectations, 81.8% of them are women.

A general description of the baseline sample in both villas is presented in Table 3. In both BDM and MB, there is a higher proportion of women than men. Most of the individuals for whom information was collected are aged between 26 and 65 years, although in MB there are more adults between 46 and 65, whereas in BDM there is a larger proportion of adults between 26 and 45. The BDM sample has a higher level of educational attainment than the MB sample, but lower than national levels, especially because of the lower proportion of individuals with more than 12 years of education (31,0% in the Chilean population [69]). Both unemployment and the proportion of people out of the labor force are higher in MB than in BDM, and the unemployment rate was higher in MB than in the country, at the time of the measurement (11.0% vs. 7.0%), according to National Employment Survey data [70].

**Table 3:** Response rates and selected characteristics of the RUCAS sample at baseline

	BDM	MB
**Sample size and retention rates**		
Baseline measurement date	April 2018	January 2019
Number of households at baseline	238	718
Response rate (% of households relative to participants or not reached in the previous wave)		
*At wave 2*	87.4%	91.9%
*At wave 3*	80.8%	90.1%
Lost to follow-up (% of households relative to baseline)		
*At wave 2*	12.6%	8.1%
*At wave 3*	23.9%	14.9%
**Sociodemographics**		
Number of individuals	682	2448
Sex		
*Male*	46.8%	47.2%
*Female*	53.2%	52.8%
Age group		
*0–15*	26.0%	25.7%
*16–25*	14.7%	18.3%
*26–45*	28.2%	23.1%
*46–65*	24.4%	28.5%
*66–99*	6.8%	4.4%
Education level according to years of study (≥ 18 years old)		
*Less than 4 years*	6.2%	8.7%
*Between 4 and 7 years*	9.9%	16.6%
*Between 8 and 12 years*	65.7%	65.7%
*More than 12 years*	18.3%	9.0%
Labor force status (≥ 15 years old)		
*Employed*	69.0%	58.9%
*Unemployed*	4.3%	7.3%
*Inactive*	26.7%	33.8%
**Dwelling (IDO tool)**		
Intervened dwellings (% relative to measured dwellings in each wave)		
*At baseline*	8.4%	0.0%
*At wave 2*	16.7%	3.6%
*At wave 3*	18.8%	8.1%
Overcrowding (≥ 2.5 persons per bedroom)	11.5%	18.3%
Presence of mold in bedrooms	37.7%	34.4%
**Perceived habitability (RUCAS survey)**		
Not satisfied with housing (overall)	34.5%	60.1%
Dwelling is perceived as “always” or “almost always” cold during winters	43.6%	71.9%
**Wellbeing and health**		
Poor reported general health (≥ 15 years old) ^a^	39.1%	46.2%
Report of medically diagnosed hypertension (≥ 15 years old)	22.2%	16.2%
Report of medically diagnosed diabetes (≥ 15 years old)	10.2%	8.1%
Depressive symptoms GHQ-12 (key informants only)	19.3%	37.6%
Current smokers (≥ 15 years old)	40.1%	43.0%
Problematic alcohol consumption (≥ 15 years old) ^b^	5.8%	12.4%
No weekly outdoor leisure time (≥ 15 years old) ^c^	45.6%	42.2%
Excessive screen time on weekends (≤ 15 years old) ^d^	59.4%	73.5%
Acute respiratory illness (preceding month)		
*At baseline (summer)*	13.2%	12.2%
*At wave 2 (winter)*	17.5%	24.2%
Gastrointestinal symptoms (preceding month)		
*At baseline (summer)*	15.0%	17.5%
*At wave 2 (winter) *	13.9%	16.9%

^a^General health was measured through a single question with seven response categories, ranging from “very bad” (1) to “very good” (7); responses lower than “good” (6) are classified as “poor”

^b^AUDIT-dimension 1 was used, with hazardous drinking defined as ≥8 points [54]

^c^Outdoor leisure time of at least 30 min

^d^ > 0 h for children under 2 years old, daily screen time; > 1 h for children 2-5 years old, daily screen time; > 2 h for children older than five, daily screen time [53]

The average number of inhabitants per dwelling is 2.9 in BDM and 3.4 in MB. Overcrowding is markedly higher in MB (18.3% vs 11.5%), but higher in both villas compared to the country (6.5% [49]). Satisfaction with the dwelling is low and perceived cold housing during winters is high in both villas, but more pronounced in MB than in BDM.

Reports of regular or poor general health, medically diagnosed hypertension and diabetes among those aged over 15 years reaches levels similar to the self-report obtained at the national level (42.3, 19.0 and 10.2%, respectively) [71]. While the prevalence of hypertension and diabetes is higher in BDM than in MB, where participants are somewhat older, the report of poor or regular general health is higher in MB than in BDM. Respondents in MB reported higher prevalence of depressive symptoms (informants only), risky alcohol consumption, and current smoking than residents of BDM. National estimates for high-risk alcohol consumption and current smoking are 11.7 and 33.4%, respectively, somewhat higher than in BDM and lower than in MB [72]. Frequencies of respiratory and gastrointestinal illness and excess of daily hours of screen exposure in children are also higher in MB than BDM. As expected, for both villas report of respiratory illness is higher in winter, while report of gastrointestinal illness is higher in summer.

Wave 3 collected information for 792 households, representing 82.8% of the baseline sample. At the household level, causes of lost to follow-up were change of place of residence out of the villa (84 households, 8.8%), study withdrawal (56, 5.9%), not reachable (23, 2.4%) and death of the informant who was the only household member (1, 0.1%). This resulted in data collection at wave 3 for 2528 individuals previously included in the baseline, whose basic sociodemographic characteristics and prevalence of chronic health conditions (diabetes and hypertension) do not significantly differ from the baseline sample (see Additional file 2).

## Discussion

Despite the abundant evidence on the relationship between housing, the built environment and health, studies on the health effects of urban regeneration in Latin America are scarce and mostly focused on slum upgrading [34]. RUCAS aims to narrow this gap by evaluating both proximal effects and more distal health effects of an urban regeneration program in two social housing neighborhoods in Chile. We expect that RUCAS will provide evidence to inform regeneration processes, highlighting the need to consider potential health effects of regeneration in the design of such interventions, following a “health in all policies” approach [73], thus contributing to make cities healthier, more equitable and socially sustainable [1–4].

Following SALURBAL’s best practices recommendations for health evaluations [74], the project was designed based on a conceptual framework developed explicitly for the assessment of a comprehensive intervention in the context of deteriorated social housing villas in Latin America, aiming to make specific predictions about the health impact of the intervention within the study time frame. The pre- and post-intervention evaluations include several validated measurements instruments, which will allow arriving at more reliable results. In the execution of the project, substantial efforts have been made to maintain a close relationship with both the communities and the professionals in charge of the intervention, to not overlook any element of the intervention that could be relevant for the evaluation of its effects and to keep high retention rates.

The complexity of such interventions offers some important research challenges for health evaluation studies [75]. So far, the intervention master plan and timeline have seen several adjustments. For example, after the first wave of measurements had taken place, changes were made to the design of the master plan in one villa, mainly in response to community demands. The timing of the intervention requires constant adjustment to exogenous factors such as occasional administrative difficulties, and broader phenomena as the *estallido social* (or social outbreak) that took place in Chile starting in October 2019, as well as the ongoing COVID-19 pandemic and associated lockdowns. Hence, the proportion of intervened dwellings in each villa for each wave has been lower than initially expected.

Given the stepped wedge design, and that the study capitalizes on within household changes over time, RUCAS has the flexibility to adapt to these external circumstances**.** In BDM, the second set of three survey waves were delayed in order to incorporate a larger number of intervened dwellings by the end of the study, given delays in the intervention. On the other hand, the incidence of the COVID-19 virus in both cities (Santiago and Viña del Mar) has been particularly high, as well as the duration of the lockdowns. Therefore, survey wave 4 has been applied via telephone, and waves 4 and 5 -conducted during winter of 2020 and summer of 2021- included questions about the health and social effects of COVID19, which is of great interest in themselves and as factors that may affect the trajectory of the study’s key health outcomes.

Another challenge of the RUCAS design lies in the collection of health and other data via a key informant. To this end, we use different approaches to measure the key health outcomes of this study, provide regular training to interviewers, and examine our results for longitudinal consistency, among others. For example, there exists comparable national estimates for some of our indicators (described above), which have proven to be similar or consistent with our sample characteristics; seasonal variations of the data are consistent with expectations; results across waves and villas are consistent in time. Similarly, for example, general health has been stratified across age and sex groups, as well as number of non-communicable diseases, with results behaving as expected in all cases (see Table 3). All these give good indication that the strategy is working well for the study aims. We have also examined thermal comfort responses, which stratified by location of the dwelling in the building (first, second and third floor) have been consistent with expectations (hottest in the top floor, coldest in the bottom floor). These perceptions will also be compared to objective measures obtained from *hygrochrons* to further validate the data about thermal comfort, given the potential role of thermal conditions in reducing respiratory health problems, as well as their centrality to the intervention in its aim to reduce fuel poverty by improving energy efficiency in the renovated buildings, favoring healthier and more sustainable social housing.

An adequate analytical design (selection of study variables in a multivariate regression model, for example) and interpretation of observed effects of the built environment and community interventions (which are for the neighborhood as a whole), as distinct from the specific effects of the dwelling intervention (which varies from dwelling to dwelling), represents another challenge. This has made been evident by our interviews and interaction with community leaderships, who have described how the intervention components intersect with each other. For example, mothers and caregivers have pointed out that they prefer their children to spend their leisure time inside because they fear insecurity and violence in the neighborhood. Thus, improvements in the neighborhood’s security could mitigate some of the effects of dwelling overcrowding, regardless of whether dwellings have been renovated or not. The selected design allows to separate these effects by comparing within household changes over time across residents of intervened and non-intervened dwellings while also accounting for time varying characteristics of the neighborhood as a whole. Interactions between dwelling and neighborhood factors can also be explored. However, the correct interpretation of the study findings will be a result of the integration of information from the study’s various sources of data.

Furthermore, qualitative data may contribute to the generation of new study hypothesis concerning, for example, potential negative effects of the intervention process when the works are disturbing to the household members, due to temporary relocation of families, or if the intervention is being largely delayed. Also, it may prove helpful in understanding whether a spillover beneficial effect may be in place in those that have not yet received the intervention but are expectant that the interventions are taking place. These insights, again, highlight the relevance of the complementarity of methods used in RUCAS, where qualitative data provides an input for the interpretation of results obtained with quantitative data, as well as for the generation of new hypotheses to be tested.

As in all longitudinal studies, lost to follow-up represents a major challenge. In the context of RUCAS, three major potential causes of lost to follow-up are: migration away from the villas, the burden of repeated surveys, and refusals to participate given the residents’ potential discomfort with the intervention, especially regarding delays in its implementation (although RUCAS has no bearing on it). Although migration away from the villas has not been frequently reported, we will conduct a short survey to explore the causes of migration and the extent to which it may be an undesired consequence of the intervention due to, for example, gentrification. To reduce whitdrawals because of the burden of participation, we will apply the full questionnaire only at baseline and at the end of the study, and short versions in the other 4 survey waves, while allowing to keep regular contact with participants and thus contributing to retention rates [65]. Finally, the project’s outreach strategy, which has permanently engaged with community leaders and partnered with the Ministry of Housing professionals, aims to ensure that community members remember and value RUCAS. During the COVID-19 pandemic this has included a close follow up of the situation in the villas. To date, lost to follow-up and its effects on sample characteristics have been satisfactorily minimized (see Additional file 2). RUCAS has been designed to measure beneficial health effects in the short term. However, some benefits are likely to manifest themselves in the long term [30]. Also, some intervention activities may have negative health effects. If so, it is possible that cases being intervened at the time of the survey report lower levels of satisfaction with the dwelling or worse health than those not yet intervened. By including an “intervention-in-progress” category in the intervention status variable we have the possibility to identify these potential negative effects.

As noted, the project is based on the concepts of urban health, following a relational approach of the link between health and place. The villas being studied are examples of the residential segregation that characterizes Latin American metropolitan cities operating within the context of neoliberalism. Sample description shows that the study populations have worse social indicators (higher unemployment [70], lower educational level and more overcrowding [49]) and health-related indicators than the general population [72]. Our approach conceives place (dwellings and villas, in this case) as socially constructed spaces which affect health, and which will be affected by the intervention. Thus, the effects of the intervention will not only be observable through changes in dwelling infrastructure, built environment, and community relations, but also through the perceptions and meanings associated with them by the inhabitants of these spaces. At the end of the intervention, living in these villas could *mean something different*. These changes, to the extent that they are also perceived as beneficial by actors outside the villas, could encourage the arrival of new inhabitants from different socioeconomic backgrounds or even an improvement in the supply of urban services, thus reducing residential segregation. The villas will be perceived as a different place in the future only if what characterizes them (the dwellings and the built and social environment) changes today. While it is likely that some health outcomes will not be significantly improved during the time frame of the RUCAS project, some proximal effects that can improve health are likely to be, such as satisfaction with the dwelling and the neighborhood, improved social relations within the villa, or the use of better-equipped recreational areas. RUCAS is an exhaustive evaluation of a comprehensive intervention, looking for the material (infrastructure) and immaterial (social relations, perceptions, uses of space, and others) effects of urban regeneration, understanding that it is in the combination of both types of effects that the improvements of the wellbeing and health of their inhabitants will be made possible.

The RUCAS project will allow the estimation of the short-term health and wellbeing effects of a comprehensive urban regeneration program implemented in two social housing villas in the periphery of two metropolitan Chilean cities. This will be accomplished through several different but complementary measurement instruments applied longitudinally between 2018 and 2021. This will allow us to capitalize on the intervention as a natural experiment. In doing so, RUCAS will contribute to the scarce evidence on the effects of formal housing and neighborhood improvement interventions on health and health determinants in Latin American cities. This research will inform the urban policy agenda of the region in the future, given that the qualitative housing deficit that characterizes Chilean social housing is a problem shared by other countries [36]. At the same time, RUCAS will allow the health and wellbeing of the population to be highlighted as a priority objective of urban and housing policy, contribute to highlight the impact of poorly designed social housing solutions, together with promoting more efficient and sustainable social housing programs [76].

## Supplementary Information

**Additional file 1.** Measurement timeframe by instrument.

**Additional file 2.** Characteristics of the RUCAS sample at baseline and remaining sample at wave 3.

## Data Availability

The data that support the findings of this study are available on request from the corresponding author AV, following RUCAS, SALURBAL and Wellcome collaboration and access policies. The data are not publicly available due to the sensitive nature of the questions asked in this study.

## Abbreviations

RUCAS: *Regeneración Urbana, Calidad de Vida y Salud* in Spanish*,* or Urban Regeneration, Quality of Life and Health in English
MINVU: *Ministerio de Vivienda y Urbanismo de Chile*, or Housing Ministry
BDM: One of the neighborhoods studied by the project, located in the city of Viña del Mar
MB: One of the neighborhoods studied by the project, located in the city of Santiago
SEREMI: *Secretaría Regional Ministerial V Región*, or Regional Ministry of Housing Office
ERU: *Equipo de Regeneración Urbana,* or Urban Regeneration Department (Ministry of Housing)
SALURBAL: *Salud Urbana en América Latina,* or Urban Health in Latin America project
IDO tool: Intra-domiciliary observation tool
SOPARC: System for Observing Play and Recreation in Communities
PARA: Physical Activity Resource Assessment
GPAQ: Global Physical Activity Questionnaire
AUDIT: Alcohol Use Disorders Identification Test
GHQ-12: 12-Item General Health Questionnaire
PHQ-2: 2-Item Patient Health Questionnaire
PEDS: Parent’s Evaluation of Developmental Status screening
EPRES: Employment Precariousness Scale
COVID-19: Coronavirus disease
