# Supplementary material for: The Regeneración Urbana, Calidad de Vida y Salud - RUCAS project: a Chilean multi-methods study to evaluate the impact of urban regeneration on resident health and wellbeing
Source: BMC Public Health. 2021 Apr 15;21:728. doi: 10.1186/s12889-021-10739-3 (PMC8047526; doi:10.1186/s12889-021-10739-3)
Supplement: Supplementary file 1 — Additional file 1. Measurement timeframe by instrument. [file 12889_2021_10739_MOESM1_ESM.pdf]

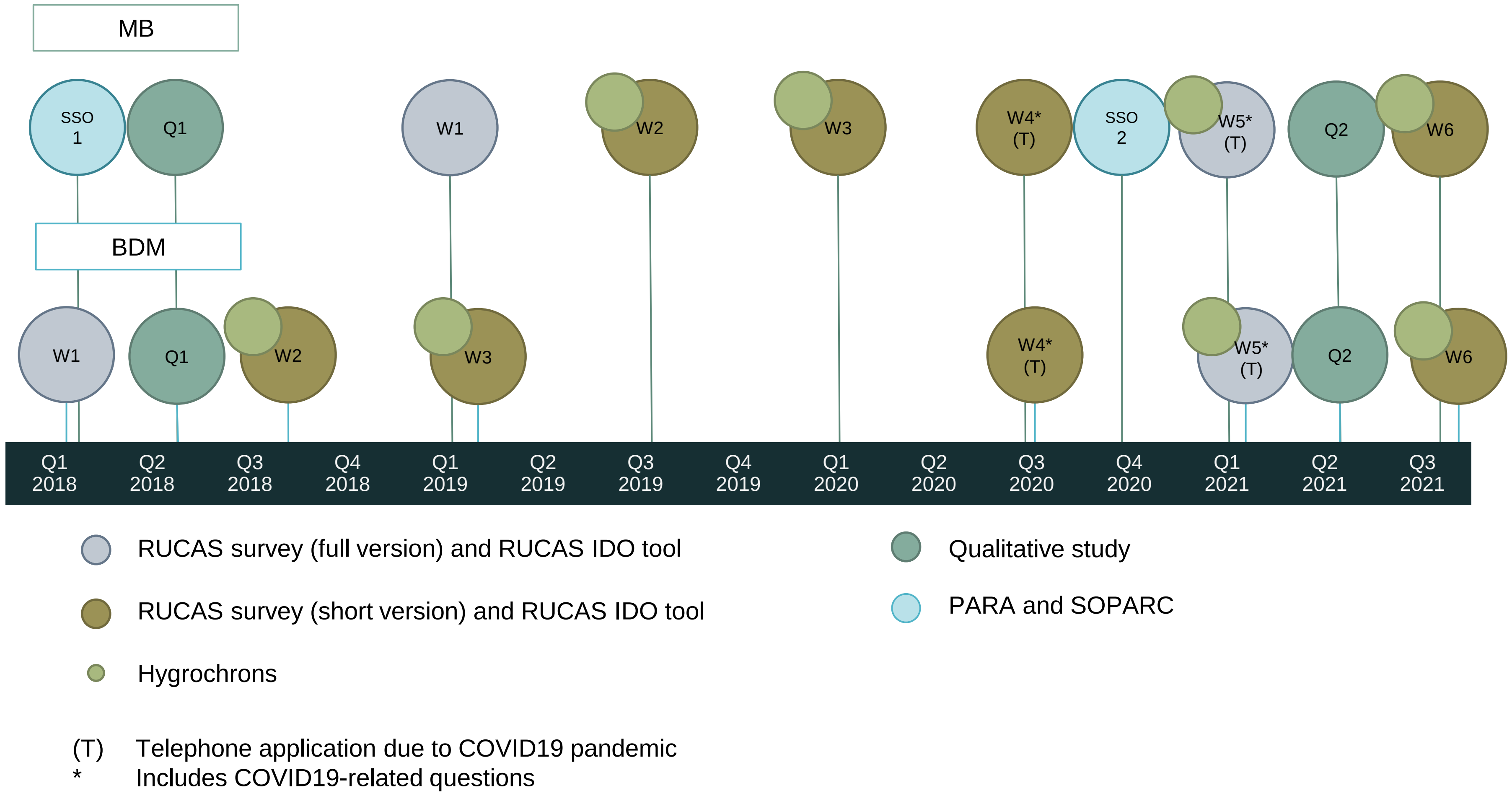
MB
SSO 1
Q1
W1
W2
W3
W4* (T)
SSO 2
W5* (T)
Q2
W6
BDM
W1
Q1
W2
W3
W4* (T)
W5* (T)
Q2
W6
Q1 2018
Q2 2018
Q3 2018
Q4 2018
Q1 2019
Q2 2019
Q3 2019
Q4 2019
Q1 2020
Q2 2020
Q3 2020
Q4 2020
Q1 2021
Q2 2021
Q3 2021
RUCAS survey (full version) and RUCAS IDO tool
RUCAS survey (short version) and RUCAS IDO tool
Hygrochrons
Qualitative study
PARA and SOPARC
(T) Telephone application due to COVID19 pandemic
* Includes COVID19-related questions
