# Supplementary material for: The Regeneración Urbana, Calidad de Vida y Salud - RUCAS project: a Chilean multi-methods study to evaluate the impact of urban regeneration on resident health and wellbeing
Source: BMC Public Health. 2021 Apr 15;21:728. doi: 10.1186/s12889-021-10739-3 (PMC8047526; doi:10.1186/s12889-021-10739-3)
Supplement: Supplementary file 2 — Additional file 2. Characteristics of the RUCAS sample at baseline and remaining sample at wave 3. [file 12889_2021_10739_MOESM2_ESM.docx]

**Additional file 2. Characteristics of the RUCAS sample at baseline and remaining sample at wave 3.**

|  | BDM | | | MB | | |
| --- | --- | --- | --- | --- | --- | --- |
|  | Baseline | Remaining | Chi-square  (p value) | Baseline | Remaining | Chi-square  (p value) |
| **Sociodemographics** | | | | | | |
| Number of individuals | 682 | 482 |  | 2448 | 2046 |  |
| Sex | | | | | | |
| *Male* | 46.8% | 45.4% | 0.204  (0.652) | 47.2% | 45.6% | 1.189 (0.276) |
| *Female* | 53.2% | 54.6% |  | 52.8% | 54.4% |  |
| Age group | | | | | | |
| *0-15* | 26.0% | 25.1% | 2.625  (0.622) | 25.7% | 26.3% | 2.084  (0.720) |
| *16-25* | 14.7% | 12.9% |  | 18.3% | 17.3% |  |
| *26-45* | 28.2% | 26.8% |  | 23.1% | 22.0% |  |
| *46-65* | 24.4% | 27.0% |  | 28.5% | 29.8% |  |
| *66-99* | 6.8% | 8.3% |  | 4.5% | 4.5% |  |
| Education level according to years of study (≥ 18 years old) | | | | | | |
| *Less than 4 years* | 6.3% | 6.9% | 0.964  (0.810) | 8.6% | 8.9% | 0.713 (0.870) |
| *Between 4 and 7 years* | 10.0% | 11.7% |  | 16.1% | 16.1% |  |
| *Between 8 and 12 years* | 64.8% | 64.2% |  | 66.2% | 66.9% |  |
| *More than 12 years* | 18.8% | 17.2% |  | 9.0% | 8.2% |  |
| **Wellbeing and health** | | | | | | |
| Report of medically diagnosed hypertension (≥ 15 years old) | 21.5% | 24.6% | 1.176  (0.555) | 16.3% | 17.6% | 1.088 (0.297) |
| Report of medically diagnosed diabetes  (≥ 15 years old) | 10.1% | 10.8% | 0.130  (0.937) | 8.1% | 8.6% | 0.278 (0.598) |
